# Health service experiences among adults with hereditary spastic paraparesis or neurofibromatosis type 1

**DOI:** 10.1002/mgg3.1399

**Published:** 2020-09-14

**Authors:** Krister W. Fjermestad, Øivind Kanavin, Livø Nyhus, Lise B. Hoxmark

**Affiliations:** ^1^ Department of Psychology University of Oslo Oslo Norway; ^2^ Frambu resource centre for rare disorders Siggerud Norway

**Keywords:** health service experiences, hereditary spastic paraparesis, HSP, neurofibromatosis type 1, NF1

## Abstract

**Background:**

Persons with rare disorders may experience poorer health services due to limited knowledge about rare disorders among health professionals. Knowledge about how persons with rare disorders perceive health services can help inform service providers to enhance their practices.

**Methods:**

We conducted a self‐report survey among adults with the rare disorders hereditary spastic paraparesis (HSP; *n* = 108; mean age 57.7 years; 54.2% females) and neurofibromatosis type 1 (NF1, *n* = 142; mean age = 50.3 years; 62.0% females). Their responses concerning perceived health experiences were compared to healthy controls from the population study HUNT‐3 (*n* = 7,312).

**Results:**

Both rare disorder groups reported lower satisfaction, trust, and participation in meetings with their general practitioner and specialist health services. More reported health problems were overall associated with poorer health service experiences.

**Conclusion:**

There is a need to identify predictors of health service experiences at the patient and health service provider levels with the aim to tighten the gap between the health experiences of patients with and without rare disorders.

## Graphical abstract

We examined the health service experiences of adults with the rare disorders, hereditary spastic paraparesis and neurofibromatosis type 1. Compared to controls, adults with rare disorders experienced poorer health services. Poorer health was associated with poorer health service experiences.

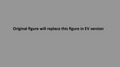

To the editor,

Persons with rare disorders, that is disorders that affect <1:2,000 individuals (Eurordis, 2020), may struggle to access and make use of health services due to limited knowledge about rare disorders among health professionals (Kerr et al., 2020). Several rare disorders also affect numerous somatic and mental domains simultaneously, and coordination between required health services may be tricky. In order to inform service providers who can work toward improving services, it is important to document how patients with rare disorders experience health services. Information about such experiences may be easier to interpret if they are examined in relation to the experiences of control persons who do not have rare disorders.

Herein, we report how 142 adults with the rare disorder neurofibromatosis 1 (NF1) and 108 adults with the rare disorder hereditary spastic paraparesis (HSP) experience health services compared to a sample of 7,312 healthy controls. NF1, an autosomal dominant genetic disorder caused by mutations or deletions of the neurofibromin gene of chromosome 17q11.2, is a multi‐organ disease characterized by susceptibility to tumor formation, changes in skin pigmentation, skeletal abnormalities, and neurological deficits, with a prevalence of 1:2,500/3,000 (Abramowicz & Gos, 2014; Williams et al., 2009). HSP, a group of neurodegenerative disorders, can be divided into pure and complex forms, with a combined prevalence of 1.8:100,000 (Ruano, Melo, Silva, & Coutinho, 2014). Pure forms of HSP are characterized by slowly progressive spastic weakness in the lower limbs, and urinary sphincter dysfunction, whereas in complex HSP, a spectrum of neurological manifestations, including ataxia, extra pyramidal signs, epilepsy, mental retardation, dementia, and peripheral nerve involvement, occurs (Harding, 1981; Salinas, Proukakis, Crosby, & Warner, 2008).

The participants were recruited through Frambu Resource Centre for Rare Disorders, one of the national advisory centers for rare disorders in Norway, and the Norwegian patient user associations for NF1 and HSP, respectively. Thus, unlike most previous reports about these disorders, we recruited the participants from nonclinical settings. The NF1 sample ranged in age from 32 to 80 years (*M* = 50.3, *SD* = 12.0; 62.0% females). The HSP sample ranged in age from 30 to 81 years (*M* = 57.7, *SD* = 11.5; 54.2% females). The controls were drawn from the third wave of the epidemiological survey “Helseundersøkelsen i Nord‐Trøndelag” (HUNT3). HUNT3 is a Norwegian survey of health conditions in which all inhabitants in Nord‐Trøndelag County aged 19 years and above were invited to participate (Krokstad et al., 2013). All three studies (the HSP study, the NF1 study, and the population control study) were approved by the respective boards for medical research ethics.

We have previously published two main reports from our study of persons with HSP and NF1 compared to controls; both showing that persons with HSP and NF1 experience considerably lower health‐related quality of life in multiple domains (Fjermestad, Kanavin, Næss, Hoxmark, & Hummelvoll, 2016; Fjermestad, Nyhus, Kanavin, Heiberg, & Hoxmark, 2018). We have also shown that those in the NF1 sample who work experience more physical and social strain in the workplace than controls (Fjermestad, 2019). In the current letter, we present previously unpublished data on health experiences. The health experience questions asked in the survey comprised eight items on experiences with the general practitioner (GP) and three items on the experiences with specialist health services. See Table 1 for overview of responses from all samples. The NF1 and HSP samples were compared to controls.

**Table 1:** Health service experiences among adults with HSP, NF1, and population controls

	HSP *n* = 108	Controls *n* = 7,312	NF1 *n* = 142
Frequency of health service use (% yes)			
Been to GP in last 12 months	60.0%**	80.2%	69.3%**
Been to specialist in last 12 months	64.4%	63.1%	61.2%
GP experience (% agree/mostly agree)			
Usually or always receive help asked for from GP	85.0%**	89.7%	81.0%**
GP has enough understanding of how your illness affects your activity level	57.1%**	51.9%	48.6%**
GP has enough understanding of your problem	76.7%**	90.8%	73.3%**
GP lets you participate in treatment decisions	80.0%*	82.8%	80.8%*
GP explains medication in a way you understand	81.4%**	88.9%	84.8%**
GP is available on the phone	56.8%*	65.7%	55.7%**
Overall ratings (1–10)			
Satisfaction with GP	7.2 (2.4)** *d* = 0.48	8.3 (2.2)	7.5 (2.4)** *d* = 0.35
Degree of trust in specialist health services	8.2 (1.5) *d* = 0.16	8.6 (3.1)	7.2 (2.4)** *d* = 0.51
Satisfaction with specialist health services	7.8 (2.0)* *d* = 0.28	8.5 (2.9)	7.3 (2.4)** *d* = 0.43

Abbreviations: GP, General practitioner; HSP, hereditary spastic paraparesis; NF1, neurofibromatosis type 1.

^*^Difference to controls is significant at the *p* < .05 level. ^**^difference to controls is significant at the *p* < .001 level.

Persons with HSP and NF1 reported fewer annual visits to the GP, lower GP availability and understanding, and overall lower satisfaction with their GP relative to controls. There was one exception to this pattern, in that more persons with HSP reported that the GP understands how the illness affects activity levels compared to controls. Whereas there were few differences between persons with HSP and controls in experiences with specialist health services; persons with NF1 generally reported lower satisfaction with specialist health services. For the scaled variables (i.e., perceived satisfaction with and trust in services), effect sizes of the differences were calculated using the formula *d* = (M_Group1_ − M_Group2_)/SD_pooled_ (Cohen, 1992). Differences were small to medium (See Table 1).

The combined GP experience items comprised a reliable scale both in the NF1 sample (*ɑ* = 0.81) and in the HSP sample (*ɑ* = 0.78). We examined this combined GP experience variable in relation to other domains in our survey, and found that health experiences were significantly and positively correlated with education in the HSP sample (*r* = .33, *p* < .001), but not in the NF1 sample. In the NF1 sample, health service experiences were significantly (all *p* < .05) and negatively associated with the level of NF1‐specific complaints (*r* = −.26), number of comorbid diseases (*r* = −.24), and gastrointestinal complaints (*r* = −.23). In the HSP sample, health service experiences were significantly (all *p* < .05) and negatively associated with HSP body impact (*r* = −.21), number of comorbid diseases (*r* = −.25), mental health problems (*r* = −.32), and gastrointestinal complaints (*r* = −.22). Thus, the overall pattern for both rare disorder groups was that more health problems were associated with poorer GP health service experiences. It is important to note that these associations may be (partly) explained by confounding variables. For example, poorer health‐related quality of life in general may lead to more negative perceptions, inability to address all problems in one visit, and frustration with lack of treatment options. Future studies should aim to disentangle patient versus practitioner variables contributing to patient experiences with health services.

Nevertheless, our findings show that there is considerable room for improvement in health services for adults with rare disorders. Although we cannot know if our results are generalizable to other rare disorders, the fact that the pattern of results are so similar for persons with two very different rare disorders implies some representativeness across rare disorders. It is perhaps unrealistic that persons with rare disorders should experience the same level of health services as patients with more prevalent (and thus more known) disorders. With >6,000 rare disorders identified to date (Eurordis, 2020), no GP could possibly keep up‐to‐date on all of these. In the HSP sample, there were fewer differences with controls in specialist health services, which may indicate that specialists display more understanding of disorder‐specific complaints than generalists—at least for some disorders. Furthermore, the fact that persons with disorders had visited the GP less often than controls may reflect the fact that these patients use their specialist as a source of annual visits or that they have more visits overall with providers and therefore do not feel the need for annual checkups.

We represent a multidisciplinary team in a specialist center for rare disorders. In our clinical experience, educating patients about their rights in the health system, including referral processes and service organization, is an important building brick toward patient empowerment. At the same time, we try to teach health professionals outside the rare disorder communities to show an open “wanting to learn” attitude in their meetings with patients with rare disorders. “*I will endeavor to find out more*” is generally a more constructive message to patients than “*I know nothing about your disorder*”. Future research should aim to identify predictors of health service experiences at the patient and health service provider levels with the aim to tighten the gap between the health experiences of patients with and without rare disorders.

## CONFLICT OF INTEREST

We all declare that we have no conflict of interest. Data are available upon request to the first author.

## AUTHOR CONTRIBUTIONS

K.W.F. analyzed the data. K.W.F, Ø.J.K., L.N., and L.B.H. planned and conducted the study. All authors read and contributed to this manuscript.
